# Facts and trends in dental antibiotic and analgesic prescriptions in Germany, 2012–2021

**DOI:** 10.1007/s00784-024-05497-6

**Published:** 2024-01-17

**Authors:** Helena Albrecht, Eik Schiegnitz, Frank Halling

**Affiliations:** 1 Department of Oral and Maxillofacial Surgery, University Medical Center of the Johannes Gutenberg-University of Mainz, Mainz, Germany; 2 Gesundheitszentrum Fulda | Praxis für MKG-Chirurgie/Plast. OP, Fulda, Germany; 3 Department of Oral and Maxillofacial Surgery, University Hospital Marburg UKGM GmbH, Marburg, Germany

**Keywords:** Prescription trends, Antibiotics, Analgesics, Germany, International comparison

## Abstract

**Objectives:**

The study aims to overview German dentists’ development of antibiotic and analgesic prescriptions from 2012 to 2021.

**Materials and methods:**

A longitudinal database analysis was performed based on the annual reports of the “Research Institute for Local Health Care Systems” (WIdO, Berlin).

**Results:**

From 2012 until 2021, dental antibiotic prescriptions fell by 17.9%. In contrast, the dental proportion of antibiotic prescriptions compared to all antibiotic prescriptions in Germany increased from 9.1 to 13.6%. Aminopenicillins enhanced their share from 35.6 to 49.4%, while clindamycin prescriptions declined from 37.8 to 23.4%. The proportion of ibuprofen prescriptions significantly increased from 60.4% in 2012 to 79.0% in 2021.

**Conclusions:**

Since 2013, the most frequently prescribed antibiotic by German dentists has been amoxicillin reaching nearly half of all dental antibiotic prescriptions in 2021. Simultaneously, the proportion of clindamycin has steadily decreased, but the level is still high compared to international data. During the past decade, ibuprofen as a first-line analgesic in German dentistry was continuously gaining in importance.

**Clinical relevance:**

Aminopenicillins have the best risk–benefit balance in dentistry, but the use of antibiotics generally must be limited only to cases of severe infections or compromised patients. Pre-existing diseases or permanent medications should always be considered when choosing an analgesic.

## Introduction

Today, analgesics and antibiotics are the most prescribed drugs by dentists. Systemic antibiotic administration and pain medication are often used to treat odontogenic infections. Dentists are confronted with odontogenic infections daily and even experienced clinicians and oral and maxillofacial surgeons face this challenge regularly. For example, a German study showed that 9.2% of all patients visited the emergency outpatient unit due to an odontogenic infection. Approximately half of these patients were treated for an abscess and the other half because of inflammatory infiltration [1]. It is common sense that surgical drainage is mandatory to achieve resolution once the abscess has formed. Antibiotic support is only indicated in special clinical situations, e.g., a compromised immune system [2, 3]. The administration of antibiotics should be limited and reduced as much as possible to prevent resistance development [4]. The most effective and tolerable antibiotic should always be used for empirical antibiotic therapy. In recent years the primary use of penicillin or amoxicillin in odontogenic infections is an international standard [5, 6]. Clindamycin can be used as an alternative drug in case of penicillin allergy [3, 7]. Unfortunately, quite a few patients claim that they are allergic to penicillin, although it turns out that only 1.0% of the allergies stated are real allergies and 99.0% are intolerances [8]. Like amoxicillin, the alternative antibiotic clindamycin shows high oral absorption, significant tissue penetration, and good penetration into bone [9, 10]. The utilization of clindamycin is enveloped by controversy due to the heightened risk of antibiotic-associated colitis and significant resistance rates [11, 12]. Besides the treatment of odontogenic infections, antibiotics are also indicated for antibiotic prophylaxis before surgical procedures. As of today, the single oral dose of 2 g of amoxicillin is recommended approximately 30 min to 1 h before the procedure [13, 14].

Selecting a suitable analgesic is also essential for a patient-centered dental treatment. The pathophysiologic pain mechanism, e.g., postoperative dental pain, nerve root inflammation, or neuropathic pain, and of course the age and morbidity of the patient influence the selection of analgesics. Because of their constrained metabolism process, the dose for children differs significantly from the adults’ treatment. Especially for elderly patients, individual risk factors like renal and hepatic diseases and comedications tend to increase and influence the choice of analgesics [15].

In scientific literature, dental prescriptions are often being analyzed either in selected fields like university clinics [16] or regional surveys [17], or in specialized sectors of health [18]. Reliable structured figures concerning the actual number and structure of prescriptions by dentists on a national scale are hardly available. Therefore, this study aims to analyze and summarize the current dental antibiotic and analgesic prescriptions in Germany for the first time, covering a whole decade (2012 – 2021). The development of the prescription of antibiotics and analgesics is analyzed, and groups of medications are compared. We focused on the absolute and relative increase of the most relevant analgesics and antibiotics in dentistry to highlight relevant trends. It also compares dental prescriptions with total antibiotic prescriptions over the investigation period.

## Materials and methods

The study is based on the data of the annual published scientific report “The Drug Prescription Report” of the WIdO, an independent research institute for local healthcare systems. The report includes all medical and dental prescriptions for members of statutory health insurance (SHI) in Germany since 2012 and the number of authorized dentists in Germany. This allows a precise retrospective analysis of the trends of antibiotic and analgesic prescriptions by German dentists over the investigation period of 10 years, starting January 1, 2012, to December 31, 2021. It includes all antibiotics and all analgesics, with more than 3.000 dental prescriptions during 2012 and 2014, respectively more than 10.000 dental prescriptions during 2015 and 2021 per year. This difference in included dental prescriptions is due to the structural change in the recording of the report.

In 2021, 73.294.342 people were members of the SHI based on a statistic from the Federal Ministry of Health, this corresponds to 88.0% of the population [19]. To analyze the trends in antibiotic and analgesic prescriptions, the unit “defined daily doses” (DDD) is used. It is a measuring unit linked to the Anatomical Therapeutic Chemical Classification (ATC). The DDD is the assumed average daily maintenance dose for a drug used for its main indication in adults [20]. There is no dependency on price, package size, and dosage form.

The pain medications included in the analysis belong to the ATC subgroups M01A (anti-inflammatory and antirheumatic products), N02AJ (opioids in combination with non-opioid analgesics), and N02B (other analgesics and antipyretics), summarized in the text as “analgesics”. The antibiotics belong to the J01 groups.

The statistical analysis and graphic illustration are made with SPSS (version 27) and Microsoft Excel (version 16.71). The Pearson correlation coefficient—which was used to determine a linear relationship between the number of SHI-insured people per dentist and their prescribing behavior, regarding antibiotic prescribing- is used to measure the strength and direction of the linear relationship between two continuous variables, ranging from -1 to 1, where 1 signifies a perfect positive linear relationship, -1 a perfect negative relationship, and 0 no linear relationship.

## Results

### Development of the number of dentists per SHI-insured person

**Fig. 1:**
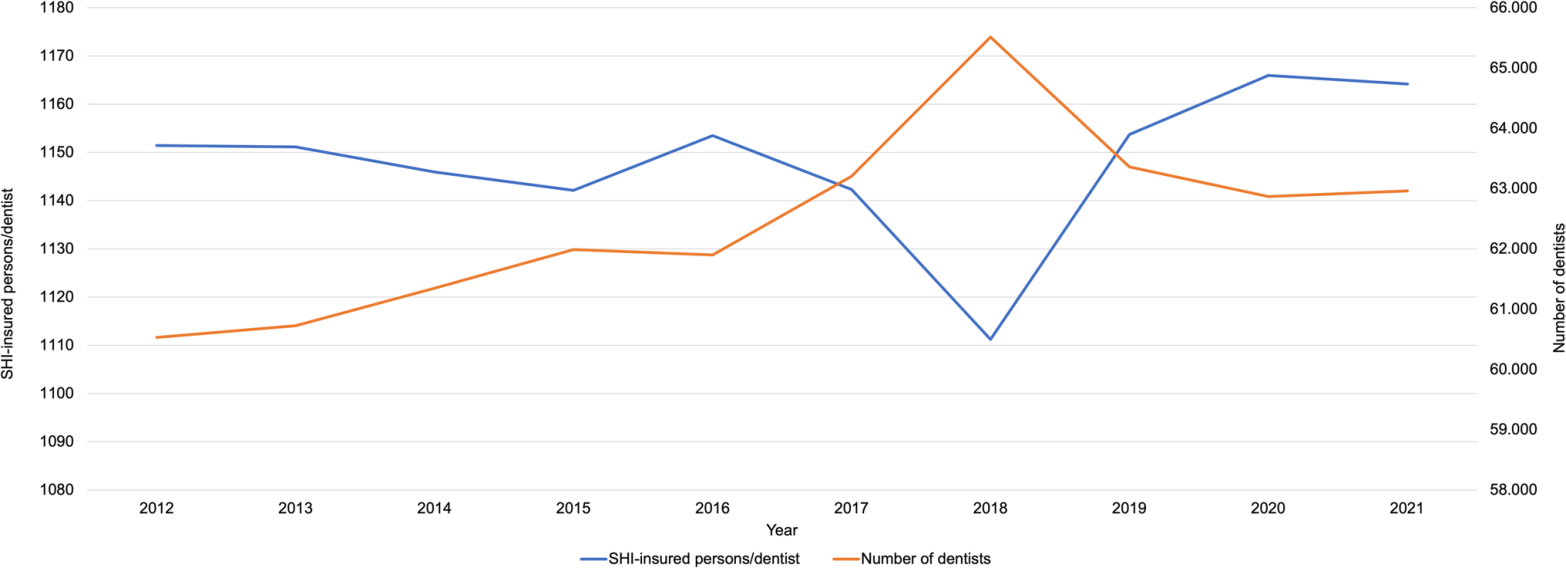
Development of the number of SHI-insured persons per dentist and the number of dentists in Germany (2012 to 2021)

During the investigation period, the absolute number of authorized dentists increased slightly likewise the number of SHI-insured persons per authorized dentist. It increased from 1151 SHI-insured persons per dentist, in 2012 (69.700.000 persons per 60.533 dentists) to 1164 in 2021 (73.300.000 persons per 62.962 dentists); resulting in a slight increase of 1.1%. Due to an increase in authorized dentists in 2018 (65.513 dentists), there was a drop in 2018. On average, there are 1148 SHI-insured persons per dentist during the investigation period. In 2020, the highest value was reached by 1167 SHI-insured persons per dentist (Fig. 1).

### The impact of the number of SHI-insured persons per dentist on their antibiotic prescribing rate

**Fig. 2:**
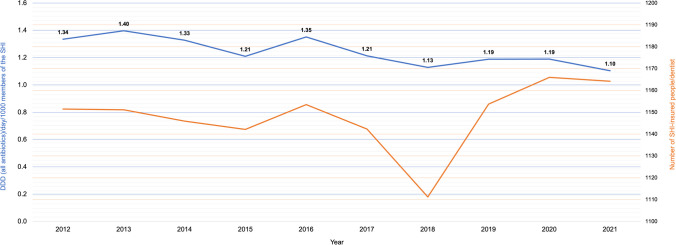
Number of SHI-insured persons per dentist during the investigation period (2012–2021) (orange), in combination with the prescription rate of antibiotics (blue). The prescription rate is stated as the DDD of all antibiotics per day per 1000 members of the SHI

This study also examined whether a correlation could be inferred for German dentists in their prescribing rate, depending on the number of SHI-insured persons per dentist. The number of SHI-insured persons per dentist did not seem to influence the antibiotic-prescribing behavior of German dentists during the investigation period (Fig. 2).

According to the Pearson correlation of 0.156, there is only a very weak positive linear relationship without significance (*p* = 0.667).

### The overall trend in the prescription of antibiotics in dental healthcare

In 2012 1.34 DDD per day per 1000 members of the SHI were prescribed, 10 years later, in 2021, only 1.10 DDD. Therefore, we found an overall decline of 17.9% in the rate of DDD of antibiotics per day per 1000 members of the SHI (Fig. 2).

**Fig. 3:**
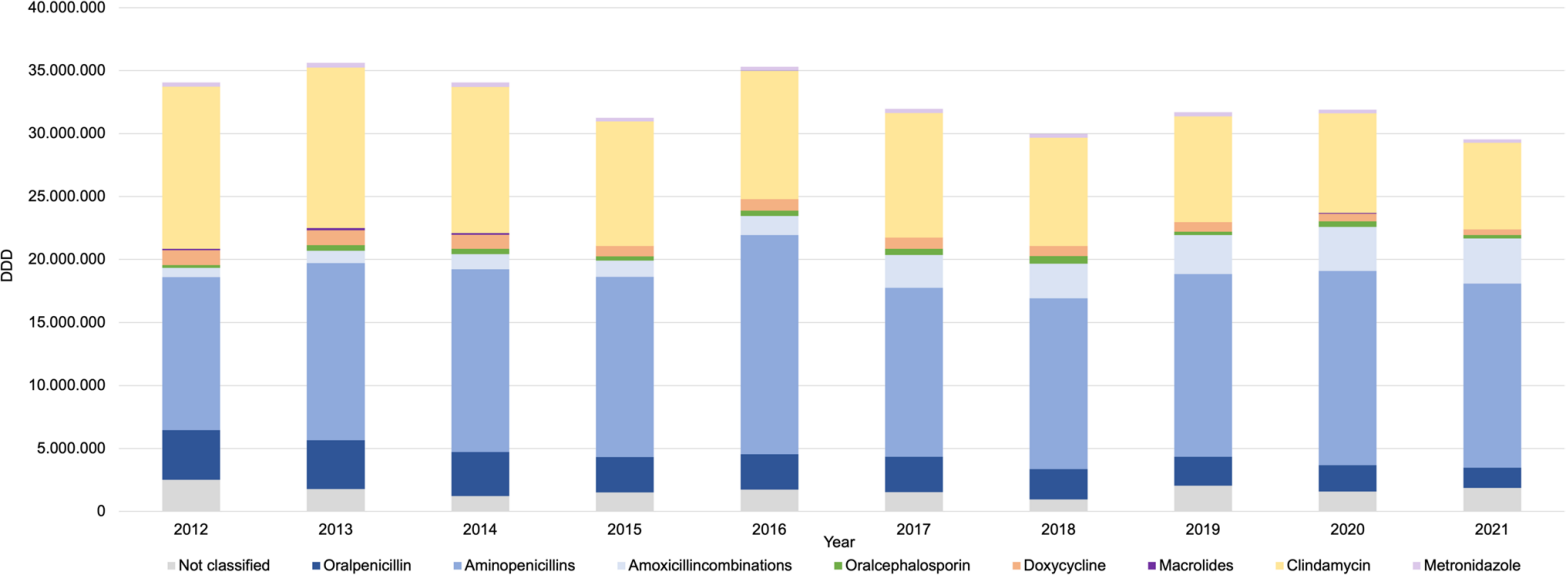
Distribution and development of antibiotic prescriptions by German dentists (2012 to 2021). Various shades of blue represent penicillin derivates, while other categories are also color-coded accordingly

Regarding the distribution of different classes of antibiotics, the group of penicillin derivatives, consisting of oralpenicillin, aminopenicillins, and amoxicillincombinations, accounts for 67.1% of all antibiotics prescribed in 2021. 2012 this share was just under half of all prescriptions (49.3%). The proportion of oralpenicillin prescriptions decreased from 11.6 to 5.4% during the observation period. Since 2013 aminopenicillins have been the most dentally prescribed antibiotic drugs in Germany, which has reached a share of nearly 50.0% in 2021. Amoxicillin is the first choice of aminopenicillin and is chosen almost exclusively by German dentists from this group, it accounts for 99.0% of all aminopenicillins in 2021. Amoxicillincombinations accounted for only 2.1% of total antibiotic prescriptions in 2012. In 2016 the proportion had approximately doubled, reaching 4.3%, and by 2021 it had even risen further to 12.2% (Fig. 3 and Table 1).

Oralcephalosporin accounts for only a very small proportion in the period under review. The share fluctuates between 0.7% and 2.0%. The prescription of doxycycline has halved since 2012, in 2021 it accounts for only an insignificant share of 1.5%. Metronidazole accounts for approximately 1.0% of all prescribed antibiotics annually and does not play a significant role in German dentistry. The number of DDDs prescribed for macrolides did not exceed 10.000 each year, so there is no indication of the number of DDDs prescribed in 2015, 2016, 2017–2019 and 2021 (Fig. 3 and Table 1).

**Table 1:** Overview of antibiotic prescriptions by German dentists (2012 to 2021)

*Year*	*2012*		*2013*		*2014*		*2015*		*2016*	
Substance	DDD	%	DDD	%	DDD	%	DDD	%	DDD	%
Penicillin derivates										
Oralpenicillin	3.950.000	11.6	3.890.000	10.9	3.500.000	10.3	2.800.000	9.0	2.800.000	7.9
Aminopenicillins	12.130.000	35.6	14.040.000	39.4	14.500.000	42.6	14.300.000	45.8	17.400.000	49.3
Amoxicillincombinations	720.000	2.1	990.000	2.8	1.200.000	3.5	1.300.000	4.2	1.500.000	4.3
Other antibiotics										
Oralcephalosporin	220.000	0.7	450.000	1.3	440.000	1.3	320.000	1.0	440.000	1.3
Doxycycline	1.200.000	3.5	1.180.000	3.3	1.100.000	3.2	840.000	2.7	900.000	2.6
Macrolides	110.000	0.3	170.000	0.5	150.000	0.4	< 10.000		< 10.000	
Clindamycin	12.870.000	37.8	12.750.000	35.8	11.600.000	34.1	9.900.000	31.7	10.200.000	28.9
Metronidazole	340.000	1.0	390.000	1.1	360.000	1.1	280.000	0.9	330.000	0.9
Not classified	2.520.000	7.4	1.770.000	5.0	1.210.000	3.6	1.510.000	4.8	1.740.000	4.9

*Year*	*2017*		*2018*		*2019*		*2020*		*2021*	
Substance	DDD	%	DDD	%	DDD	%	DDD	%	DDD	%
Penicillin derivates										
Oralpenicillin	2.800.000	8.8	2.400.000	8.0	2.300.000	7.3	2.100.000	6.6	1.600.000	5.4
Aminopenicillins	13.400.000	41.9	13.600.000	45.4	14.500.000	45.7	15.400.000	48.3	14.600.000	49.4
Amoxicillincombinations	2.600.000	8.1	2.700.000	9.0	3.100.000	9.8	3.500.000	11.0	3.600.000	12.2
Other antibiotics										
Oralcephalosporin	500.000	1.6	610.000	2.0	260.000	0.8	460.000	1.4	260.000	0.9
Doxycycline	900.000	2.8	800.000	2.7	760.000	2.4	600.000	1.9	450.000	1.5
Macrolides	< 10.000		< 10.000		< 10.000		70.000	0.2	< 10.000	
Clindamycin	9.900.000	31.0	8.600.000	28.7	8.400.000	26.5	7.900.000	24.8	6.900.000	23.4
Metronidazole	320.000	1.0	330.000	1.1	350.000	1.1	300.000	0.9	250.000	0.9
Not classified	1.540.000	4.8	950.000	3.2	2.040.000	6.4	1.570.000	4.9	1.870.000	6.3

**Fig. 4:**
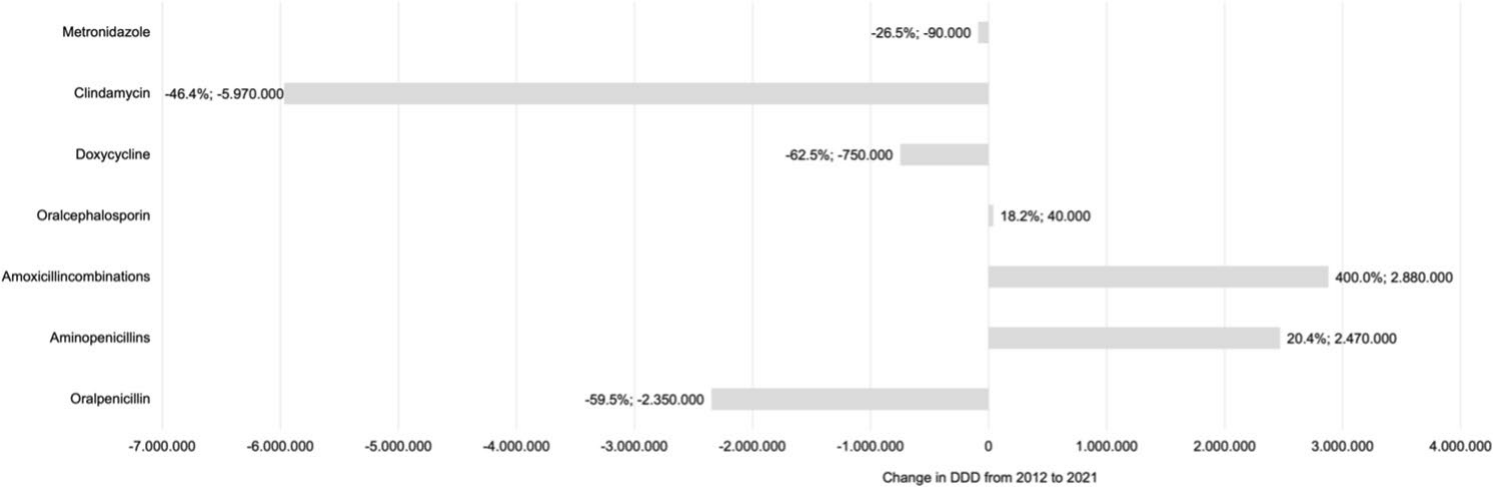
Change in the number of DDD each antibiotic prescribed from 2012 to 2021 including percentage increase or decrease

A comprehensive overview of the prescription trends of each antibiotic by German dentists during the study period from 2012 to 2021 is provided in Fig. 4. The largest percentage increase over the 10 years is due to amoxicillincombinations (400.0%). The largest percentage decrease was observed in doxycycline (-62.5%) (Fig. 4).

**Fig. 5:**
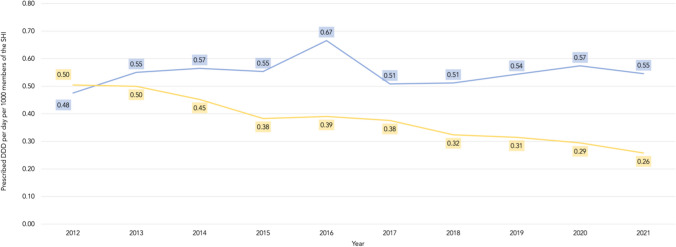
Comparison between the development of the dental prescription rate of clindamycin and aminopenicillins by German dentists in the investigation period (2012 to 2021)

Referring to the prescribed DDD per day per 1000 members of the SHI of clindamycin and aminopenicillins as the most important dental antibiotics in Germany both started from a comparable level in 2012 (0.5 vs. 0.48). During the following decade, we could find a contrary development of their prescriptions. The prescribed DDD of clindamycin nearly fell by half to 0.26 DDD, whereas aminopenicillins increased by 14.6% to 0.55 DDD in 2021 (Fig. 5).

### Share of dental antibiotic prescriptions 2012–2021

Since 2012, the number of antibiotic prescriptions (DDD) in Germany in total (medicine and dentistry) has decreased by 41.6% (Table 2). In medicine, the reduction rate was 44.4% during the decade. Remarkably, the reduction of dental antibiotic prescriptions was considerably smaller which means only 13.5% during the investigation period. Accordingly, the percentage of dental prescriptions in relation to all antibiotic prescriptions in Germany has increased from 9.1% In 2012 to 13.6% In 2021.

**Table 2:** Share of dental prescriptions of the total amount of prescribed DDD of antibiotics (2012 to 2021)

*Year*	*Total prescriptions*	*Medical prescriptions*	*Dental prescriptions*	*Dental share*
	DDD in M	DDD in M	DDD in M	*(of all prescriptions)*
2012	372.5	338.4	34.1	9.1
2013	401.8	366.2	35.6	8.9
2014	374	339.9	34.1	9.1
2015	372.6	341.3	31.3	8.4
2016	373	337.7	35.3	9.4
2017	336.8	304.8	32.0	9.5
2018	316	286	30.0	9.5
2019	308.8	277.1	31.7	10.3
2020	244	212.1	31.9	13.1
2021	217.6	188.1	29.5	13.6
*Percentage Reduction* (2012 to 2021)	41.6%	44.4%	13.5%	

### Development of prescription of analgesics

**Fig. 6:**
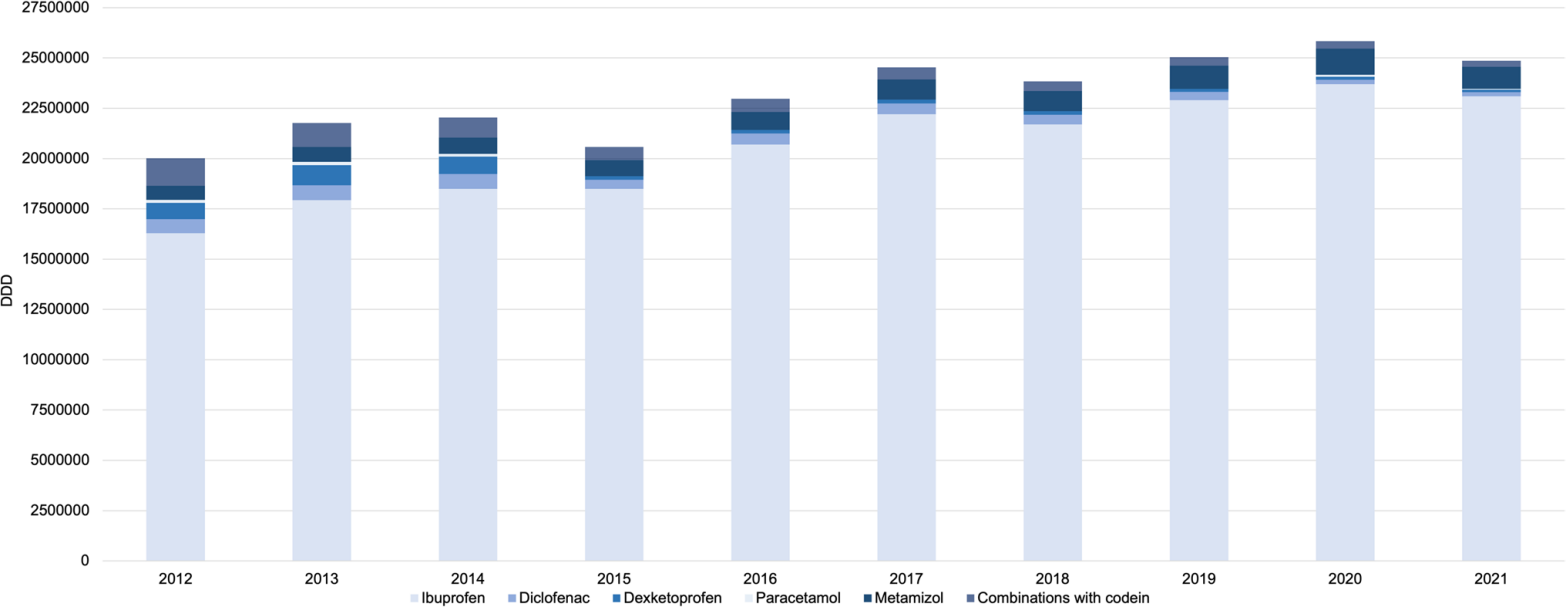
Distribution and development of analgetic prescriptions by German dentists (2012 to 2021)

When considering the prescription rate of analgesics and anti-inflammatories, it is noticeable that there is a slightly increasing trend of 8.4% over the observation period, but the distribution has changed significantly over the years (Fig. 6).

Ibuprofen has almost completely displaced the other NSAIDs and has taken a dominant share. In 2012 the share of NSAIDs was 65.9%, while in 2021 it has already reached 80.1%. In this year other NSAIDs like diclofenac and dexketoprofen were rarely prescribed and accounted for only 1.0% of all analgetic prescriptions (Table 3).

The prescription of metamizole has almost doubled, so in the year 2021, metamizole already accounts for 3.8% of all analgesic prescriptions. Combinations with codeine have significantly decreased from 5.1 to 1.0% in 2021. Unclassified analgesics comprise a smaller share than ten years ago (25.9% vs. 15.0%) (Table 3).

**Table 3:** Absolute number of DDD of prescribed analgesics and antiphlogistics and their share (2012 to 2021)

Year	2012		2013		2014		2015		2016	
Substance	DDD	%	DDD	%	DDD	%	DDD	%	DDD	%
NSAID										
Ibuprofen	16.290.000	60.4	17.930.000	62.5	18.500.000	66.6	18.500.000	72.2	20.700.000	72.7
Diclofenac	700.000	2.6	740.000	2.6	740.000	2.7	450.000	1.8	540.000	1.9
Dexketoprofen	800.000	3.0	1.000.000	3.5	860.000	3.1	180.000	0.7	180.000	0.6
Others										
Paracetamol	160.000	0.6	170.000	0.6	140.000	0.5	< 10.000		< 10.000	
Metamizole	690.000	2.6	740.000	2.6	800.000	2.9	790.000	3.1	890.000	3.1
Comb. with codeine	1.370.000	5.1	1.190.000	4.2	1.000.000	3.6	660.000	2.6	660.000	2.3
Not classified	6.980.000	25.9	6.930.000	24.2	5.730.000	20.6	5.030.000	19.6	5.500.000	19.3

Year	2017		2018		2019		2020		2021	
Substance	DDD	%	DDD	%	DDD	%	DDD	%	DDD	%
NSAID										
Ibuprofen	22.200.000	72.8	21.700.000	74.8	22.900.000	73.5	23.700.000	71.9	23.100.000	79.0
Diclofenac	540.000	1.8	480.000	1.7	410.000	1.3	220.000	0.7	200.000	0.7
Dexketoprofen	190.000	0.6	170.000	0.6	160.000	0.5	150.000	0.5	120.000	0.4
Others										
Paracetamol	< 10.000		< 10.000		< 10.000		90.000	0.3	40.000	0.1
Metamizole	1.000.000	3.3	1.000.000	3.5	1.150.000	3.7	1.300.000	3.9	1.100.000	3.8
Comb. with codeine	600.000	2.0	480.000	1.6	420.000	1.4	380.000	1.2	300.000	1.0
Not classified	5.950.000	19.5	5.180.000	17.9	6.130.000	19.7	7.130.000	21.6	4.386.000	15.0

During the observation period the relative share of ibuprofen among all dental pain medications has considerably increased. In 2012 ibuprofen accounted for 60.4% of the total, whereas in 2021 the share reached 79.0% of all prescribed pain medications (Table 3).

**Fig. 7:**
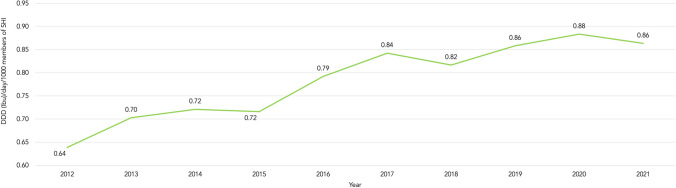
Development of the prescription rate of Ibuprofen (2012 to 2021)

Simultaneously, the prescription rate of ibuprofen steadily increased with slight fluctuations. In 2012, 0.64 DDD of ibuprofen were prescribed per day per 1000 members of the SHI, while in 2021 the prescription rate had already risen to 0.86 DDD (+ 34,5%) (Fig. 7).

## Discussion

This research is the initial analysis of the prescription patterns of German dentists for antibiotics and analgesics over the course of ten years. It provides a detailed *overview* of the usage trends of specific antibiotics and analgesics, offering extensive data that reflects their development.

Regarding antibiotics, the most significant advancements were made in prescribing two types of antibiotics: aminopenicillins and clindamycin. Aminopenicillins—especially amoxicillin, with a share of 99.0% of all prescribed aminopenicillins in Germany—have taken over the role that clindamycin held a decade ago. The prescription of clindamycin has steadily decreased, partly due to the increasing prevalence of resistance [4], making this development a positive change. An English study about adverse reactions to antibiotics revealed a complete lack of fatal reactions but 22.62 nonfatal reactions per million amoxicillin prescriptions. For clindamycin, there were 13 fatal reactions and 149 nonfatal reactions per million prescriptions. Most clindamycin adverse reactions were *Clostridioides difficile* infections [21]. Clindamycin should therefore be used only when penicillin allergy is proven and not just claimed by the patient. The use of clindamycin for endocarditis prophylaxis, for example, is already completely discouraged in the US, as the American Heart Association mentioned in a scientific statement in April 2021 [14]. Nevertheless, clindamycin remains the second most prescribed antibiotic in Germany. In 2012 the amount of clindamycin prescribed was 0.50 DDD per day per 1000 members of the SHI. This was compared to 0.48 DDD of aminopenicillins prescribed in the same year. In 2021 half as many DDD of clindamycin (0.26 DDD per day per 1000 members of the SHI) were prescribed compared to aminopenicillins (0.55 DDD per day per 1000 members of the SHI). In a recent German study, it was published that dental prescriptions of clindamycin still made up 56.0% of all clindamycin prescriptions in primary care in 2021 [22]. Despite the decline of dental prescriptions in Germany during the last decade the current share of clindamycin is still significantly higher than in other countries, for example, in England, Norway, British Columbia, and Canada [23].

When comparing the number of antibiotics prescribed for medical and dental purposes, we noticed a concerning trend that might be linked to the COVID-19 pandemic. In 2012 dental prescriptions accounted for 9.1% of all prescriptions. Although the number of antibiotic prescriptions has decreased since then, the proportion of dental prescriptions increased to 13.6% in 2021, representing almost a 50.0% increase over ten years. 2020 there was a significant jump in the share of dental prescriptions from 10.3 to 13.1%. This could be due to the measures taken to prevent the spread of COVID-19, which may have led to a decrease in general practitioners’ antibiotic prescriptions but not in dentists’. Some measures were still in place in Germany until the end of the investigation period, and people remained cautious, which may explain the ongoing increase in dental prescriptions. However, further studies are necessary to support this claim.

Data from a study from 2013 to 2016 in Australia has shown a decrease in the total amount of antibiotics prescribed by dentists, but there has been an increase of 11.2% in the prescription of amoxicillin/clavulanic acid. Like in Germany, amoxicillin was the most dispensed antibiotic, accounting for a share of approximately 65.0%, while phenoxymethylpenicillin accounted for only 1.4% of prescriptions in 2016 [24]. A cohort study in the US for the 2013 to 2015 citation period revealed that antibiotic prescribing rates remained stable over the investigation period [25]; by comparison, in Germany during the same period overall relative prescribing (DDD/day/SHI-insured persons) decreased by 13.6%. A current Italian study shows that local dentists mainly prescribe macrolides as an alternative medication in cases of penicillin allergy. Macrolides were prescribed in 85.0% of the cases. In contrast to Italy, macrolides play no role in the German dental prescribing routine. Lincosamides, such as clindamycin, accounted for a much lower proportion than in Germany (4.2% vs. 23.4%) [26]. A recent study published in 2020 from Colombia indicates that the surveyed dentists almost exclusively prescribe amoxicillin (80.4%) as the first-choice antibiotic. The second-choice antibiotics included clindamycin (43.6%), the macrolides azithromycin and erythromycin (56.7%), cephalexin (18.4%), and amoxicillin/clavulanic acid combination (15.7%) [27]. In a current study from *Brazil*, the highest number of dental prescriptions was for amoxicillin, followed by macrolides like azithromycin [28]. Therefore, macrolides being antibiotics with solely bacteriostatic effects in contrast to the bactericidal penicillins, significantly impact Italy, Columbia, and Brazil more than Germany.

Heavy workload is often cited as a reason for frivolous antibiotic prescribing in current literature [29–31]. In this study, no significant correlation could be shown between the dentists' workload in terms of the number of patients per dentist, and the number of antibiotics prescribed.

There are only a few current studies from other countries regarding the dental prescription of analgesics. However, certain tendencies can be inferred. For example, the use of opioids is known to play a significant role in the US, while they are rarely prescribed in Germany [32]. From a study conducted in Guangzhou, China, in 2020 it emerged that dentists most commonly prescribe paracetamol and diclofenac as analgesics [33]. Over the decade, the development of analgesic prescriptions shows that ibuprofen drives most other analgesics off the market. The trend of increasing ibuprofen prescriptions observed in former studies has continued [34]. At the same time, we know that NSAIDs can be associated with gastrointestinal and cardiovascular adverse events [34–36]. The appropriate NSAID should be tailored to the individual risk profile, especially with regard to elderly people [36]. It is striking that paracetamol is hardly prescribed. However, it should be mentioned that both ibuprofen and paracetamol are commonly purchased and consumed by patients without a prescription. A recent German study showed that 65.0% of respondents reported using over-the-counter drugs frequently or occasionally [37]. Since independent purchasing may be more cost-effective for patients without a prescription, analgesic prescriptions only provide insight into using analgesics and do not represent an absolute value.

Despite the fact, that there are no specific dental indications for metamizole except acute severe pain after surgery [38], the dental prescriptions of metamizole have significantly increased over the past decade. However, the low share (3.8%) in the dental sector is remarkable, notably against the background that metamizole is generally one of the most commonly prescribed drugs in Germany [39]. Nevertheless, dentists’ increased metamizole prescription must be observed critically due to its serious side effects [40]. It is important to give special mention to the potentially deadly metamizole-associated agranulocytosis [41]. The incidence of metamizole-induced agranulocytosis is controversial, but the risk will likely be limited with short-term postoperative use in this selected group of patients [42]. Although firm evidence is lacking, metamizole may be safer for the upper intestinal tract and kidneys than other NSAIDs. It could alternatively be used in patients with an increased risk for stomach or renal problems [42].

The benefits of our study are their high reliability because of a board database, which includes all SHI-insured people, representing nearly 90.0% of the German population. Accordingly, a general statement can be made about using antibiotics and analgesics in German dentistry. There are also only a few unreported cases because the group “others” contains just about 5.0% in antibiotics and about 20.0% in analgesics since many combinations are prescribed here. Limitations of the study are a dearth of information on the dosage, frequency, and duration of administration, the combinations of antibiotics used, and the reasons for individual prescriptions. Additionally, no reliable data is available on the indications of antibiotic prescriptions or the prescribing practices of specialized dental practitioners. As mentioned above, the number of prescribed analgesics gives only insight and is not transferable to the total consumed analgesics because of the high rate of unrestricted over-the-counter analgesics. Therefore, it is imperative to conduct further investigations on these pressing issues.

## Conclusion

Since 2012, the share of antibiotics prescribed by dentists in relation to the total amount of antibiotic prescriptions in Germany has increased from 9.1 to 13.6%. Amoxicillin has become the primary antibiotic since 2013, replacing clindamycin due to a rising trend. Nevertheless, the percentage of clindamycin prescriptions remains high, accounting for one-quarter of all antibiotics prescribed. Like in Germany amoxicillin is often the primary antibiotic prescribed in dentistry across numerous countries. In contrast to other countries macrolides play no role in German dentistry.

Regarding pain relief medication ibuprofen is becoming increasingly popular and has surpassed all other analgesics in Germany. In 2021, Ibuprofen prescriptions accounted for 79% of all dental analgetic prescriptions. Because of a high portion of analgesics sold without a prescription the real consumption of painkillers in the dental sector remains unclear.
